# The Association between Troponin-I Clearance after the Return of Spontaneous Circulation and Outcomes in Out-of-Hospital Cardiac Arrest Patients

**DOI:** 10.31083/j.rcm2501024

**Published:** 2024-01-15

**Authors:** Dong Hun Lee, Byung Kook Lee, Seok Jin Ryu

**Affiliations:** ^1^Department of Emergency Medicine, Chonnam National University Hospital, 61469 Gwangju, Republic of Korea; ^2^Department of Emergency Medicine, Chonnam National University Medical School, 61469 Gwangju, Republic of Korea

**Keywords:** troponin, cardiac arrest, prognosis, clearance

## Abstract

**Background:**

Elevated levels of troponin-I (TnI) are common in out-of-hospital cardiac arrest (OHCA) patients. However, studies evaluating the prognostic value of TnI clearance in OHCA patients are lacking. We aimed to examine how TnI clearance (TnI-C) differed according to the neurological outcome group and mortality group at 6 months.

**Methods:**

This retrospective observational study involved adults (≥18 years) who were treated for an OHCA between January 2019 and December 2022. The TnI-Cs were calculated for days 1 to 2 (TnI-C1st) and 2 to 3 (TnI-C2nd) after the return of spontaneous circulation (ROSC). The primary outcome was a poor neurological outcome at 6 months, defined by cerebral performance categories 3, 4, and 5. The secondary outcome was 6-month mortality.

**Results:**

A total of 227 patients were included. A poor neurological outcome and mortality at 6-months were reported in 150 (66.1%) and 118 (52.0%) patients, respectively. The TnI-C1st was significantly lower in patients with a poor outcome compared with good outcome patients (neurological outcome at 6 months, 54.4% vs. 42.3%; 6-month mortality, 52.1% vs. 42.7%, respectively). In the multivariable analyses, a TnI-C1st <50% was associated with a poor neurological outcome (odds ratio [OR] 2.078, 95% confidence interval [CI] 1.080–3.995, *p* = 0.028) and mortality (OR 2.131, 95% CI 1.114–4.078, *p* = 0.022) at 6 months.

**Conclusions:**

After ROSC, TnI-C1st <50% was associated with a poor neurological outcome and mortality at 6 months in OHCA patients.

## 1. Introduction

Although progress in post-cardiac arrest management has improved clinical outcomes, including in targeted temperature management (TTM) and goal-directed therapies, the prognosis for most patients suffering out-of-hospital cardiac arrest (OHCA) remains poor [1, 2, 3]. Guidelines recommend a multimodal prognostic approach in cardiac arrest survivors to promote the patient’s prognostication [1]. Of the available prognostic tools, biomarkers have the advantage of being minimally affected by sedatives.

Troponin testing has high sensitivity and specificity for myocardial injury, while elevated troponin levels are common after OHCA since myocardial injury occurs [4, 5]. However, in several studies, the initial troponin level did not reflect the prognosis of OHCA patients [5, 6, 7]. Conversely, lactate may also be related to myocardial ischemia, while lactate clearance was related to OHCA prognosis in previous studies [8, 9, 10]. While troponin clearance has been associated with patient prognosis in several severe diseases [11, 12], studies evaluating troponin clearance for the prognosis of OHCA patients are lacking.

We hypothesized that a lower troponin clearance after the return of spontaneous circulation (ROSC) is related to a poor neurological outcome or mortality in OHCA patients. Therefore, we aimed to investigate any differences in troponin clearance between neurological outcome groups and mortality groups 6 months after cardiac arrest.

## 2. Methods

### 2.1 Study Design and Population

This retrospective observational study involved adult (age ≥18 years) OHCA patients treated with TTM at a University-affiliated tertiary hospital between January 2019 and December 2022, and it was approved by the Chonnam National University Hospital Institutional Review Board (CNUH-2023). Patients’ troponin-I (TnI) levels were continually measured up to 3 days after ROSC. We excluded patients who were younger than 18 years, lacked TnI measurements, or had terminated TTM due to transfer or death.

### 2.2 Study Protocol

Comatose cardiac arrest patients underwent TTM according to the written guideline-based protocol. Patients were cooled to a target temperature of 32–36 °C, avoiding a fever >37.5 °C. Target temperatures were maintained for 24 hours using a surface cooling device (Arctic Sun™ Energy Transfer Pads, Becton, Dickinson and Company (BD), Franklin Lakes, NJ, USA). Propofol and remifentanil were administered during TTM to achieve sedation and analgesia.

Data related to the following variables were obtained from each patient’s hospital records: age, sex, body mass index, preexisting illnesses, witnessed collapse, bystander cardiopulmonary resuscitation (CPR), first on-scene monitored rhythm, time from sudden cardiac arrest to ROSC, and calculated Sequential Organ Failure Assessment (SOFA) score within 24 hours of admission. Serum laboratory values, such as lactate and glucose levels, and results from arterial blood gas analyses, such as partial pressure of oxygen (${\mathrm{PaO}}_{2}$) and carbon dioxide (${\mathrm{PaCO}}_{2}$), were obtained upon admission to the emergency department.

Blood samples were drawn to measure the TnI levels between 6 and 12 (T1), 12 and 18 (T2), 24 and 30 (T3), and 48 and 54 (T4) hours after ROSC. Additional TnI measurements were taken according to the physician’s discretion. The serum TnI measurement method used in this study was the high-sensitivity one-step immunoassay with an analytical range of 0 to 0.05 ng/mL (ADVIA Centaur® XP/XPT, Siemens AG, Munich, Germany). Peak TnI levels were expressed according to the time from ROSC as follows: before 24 (TnI1st); 24 to 48 (TnI2nd); 48 to 72 (TnI3rd) hours. The TnI clearances (TnI-C) were calculated as ((TnI1st – TnI2nd)/TnI1st) × 100, and they were expressed as TnI-C1st and TnI-C2nd. Troponin is continuously leaked from a necrotic myocardium; therefore, its apparent half-life after a myocardial infarction is about 24 hours [13]. Thus, we expressed TnI-C as a nominal variable when it was less than 50%.

Neurological outcomes and mortality were evaluated 6 months after ROSC through structured telephone interviews with patients or their caregivers [14]. A neurological outcome was evaluated via the cerebral performance category (CPC) scale (CPC 1, good performance; CPC 2, moderate disability; CPC 3, severe disability; CPC 4, vegetative state; or CPC 5, brain death or death) [15]. The primary outcome was a poor neurological outcome at 6 months, defined as CPC 3, 4, or 5. The secondary outcome was mortality at 6 months.

### 2.3 Statistical Analysis

We presented the categorical variables as frequencies and percentages, and we illustrated continuous variables as the mean ± standard deviation or the median and interquartile range, depending on the Shapiro–Wilk test results. Categorical variables were compared using the χ^2^ test, while continuous variables were compared between the groups using independent *t*-tests or Mann–Whitney U tests.

We performed a multivariable logistic regression analysis to identify the association between TnI-C and poor neurological outcome or mortality at 6 months. Variables with *p*-values < 0.20 on univariate comparisons were included in the multivariable regression model. We used a backward stepwise approach that sequentially eliminated variables with *p*-values > 0.10 to build a final adjusted regression model. The variables for TnI levels were included in the final model. The results of the logistic regression analysis are presented as the odds ratio (OR) and 95% confidence intervals (CIs). All analyses were conducted using PASW Statistics for Windows, version 18.0 (SPSS Inc., Chicago, IL, USA). Statistical significance was defined as *p* < 0.05 (two-sided).

## 3. Results

### 3.1 Patient Characteristics

**Fig. 1.**
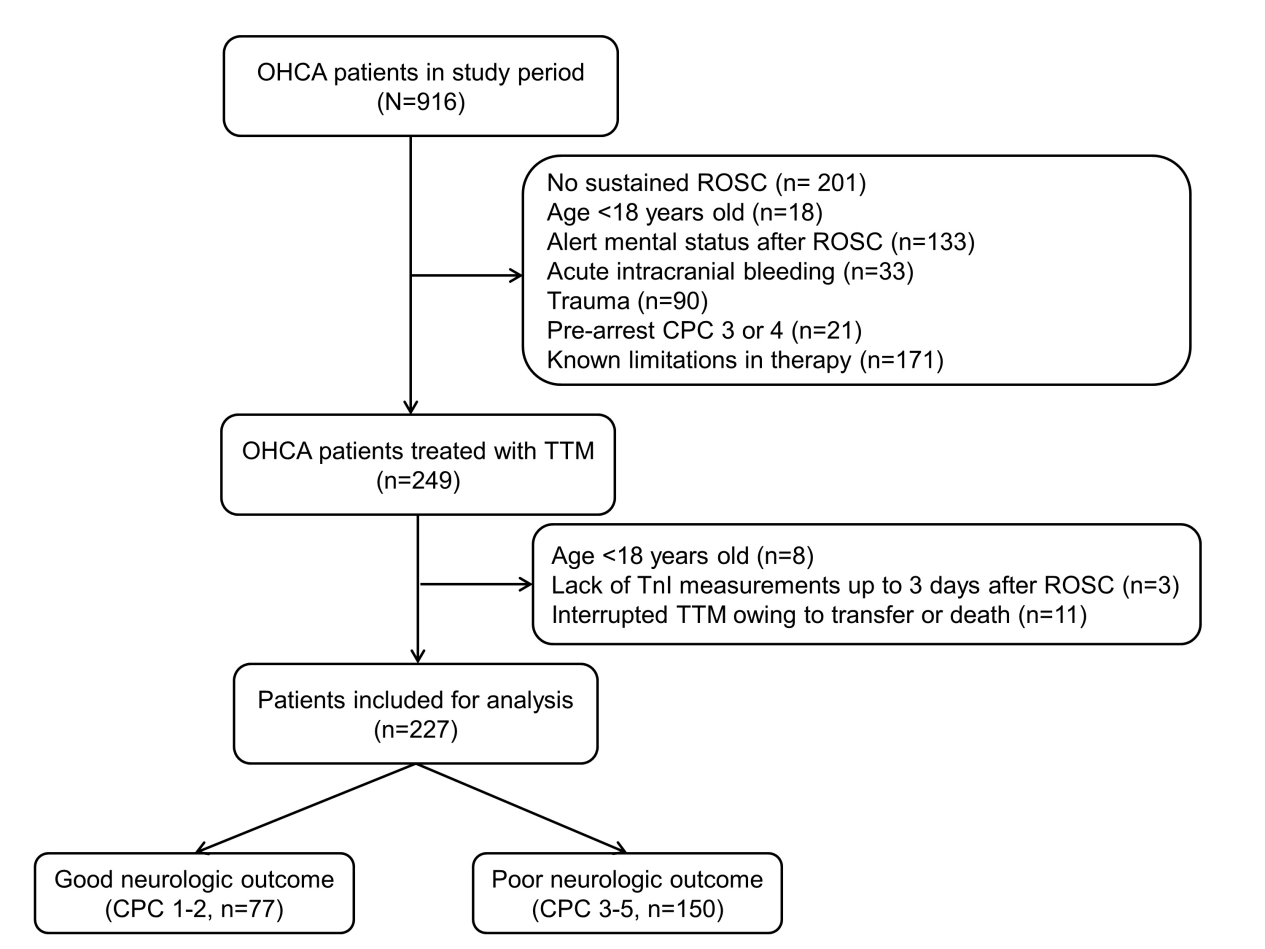
**Schematic diagram showing the number of out-of-hospital cardiac arrest patients**. OHCA, out-of-hospital cardiac arrest; ROSC, return of spontaneous circulation; CPC, cerebral performance category; TTM, targeted temperature management.

During the study period, 249 OHCA patients were treated with TTM. Fig. 1 provides an overview of the study. A total of 227 patients were involved in the study. Their median age was 62.0 (50.0–71.0) years, and the median downtime was 27.0 (17.0–43.0) minutes (Table 1). Of the patients included, 150 (66.1%) had a witnessed collapse, 138 (60.8%) received bystander CPR, 93 (41.0%) had a shockable monitored rhythm, and 135 (59.5%) had a cardiac etiology. Poor neurological outcomes and death at 6 months were reported in 150 (66.1%) and 118 (52.0%) patients, respectively.

**Table 1.** **Comparisons of baseline characteristics according to neurological outcomes and mortality at 6 months**.

Variables		Total (n = 227)	Neurological outcomes at 6 months			Mortality at 6 months		
			Good (n = 77)	Poor (n = 150)	*p*	Survivors (n = 109)	Non-survivors (n = 118)	*p*
Demographics								
	Age, years	62.0 (50.0–71.0)	57.0 (44.5–66.5)	66.0 (52.8–75.0)	<0.001	59.0 (47.0–69.5)	66.5 (51.8–75.0)	<0.001
	Male, n (%)	165 (72.7)	57 (74.0)	108 (72.0)	0.867	85 (78.0)	80 (67.8)	0.116
	Body mass index, ${\mathrm{kg/m}}^{2}$	23.5 (21.1–23.5)	24.2 (22.5–26.4)	23.1 (20.6–25.1)	0.003	23.7 (21.6–25.9)	23.4 (20.8–25.8)	0.003
Preexisting illness, n (%)								
	Coronary artery disease	42 (18.5)	15 (19.5)	27 (18.0)	0.927	21 (19.3)	21 (17.8)	0.909
	Congestive heart failure	9 (4.0)	3 (3.9)	6 (4.0)	1.000	4 (3.7)	5 (4.2)	1.000
	Hypertension	113 (49.8)	27 (35.1)	86 (57.3)	<0.002	47 (43.1)	66 (55.9)	0.072
	Diabetes	68 (30.0)	12 (15.6)	56 (37.3)	<0.001	26 (23.9)	42 (35.6)	0.074
	Renal impairment	21 (9.3)	3 (3.9)	18 (12.0)	0.080	7 (6.4)	14 (11.9)	0.236
Cardiac arrest characteristics								
	Witnessed collapse, n (%)	150 (66.1)	58 (75.3)	92 (61.3)	0.050	79 (72.5)	71 (60.2)	0.069
	Bystander CPR, n (%)	138 (60.8)	52 (67.5)	86 (57.3)	0.178	74 (67.9)	64 (54.2)	0.049
	Shockable rhythm, n (%)	93 (41.0)	59 (76.6)	34 (22.7)	<0.001	71 (65.1)	22 (18.6)	<0.001
	Cardiac etiology, n (%)	135 (59.5)	66 (85.7)	69 (46.0)	<0.001	84 (77.1)	51 (43.2)	<0.001
	Time to ROSC, min	27.0 (17.0–43.0)	19.0 (14.0–27.0)	31.5 (21.5–47.3)	<0.001	20.0 (14.0–35.0)	31.5 (22.0–48.0)	<0.001
Characteristics after ROSC								
	Lactate, mmol/L	8.2 (5.0–11.4)	6.4 (6.2–9.6)	9.4 (6.0–12.2)	<0.001	6.7 (4.1–9.5)	9.6 (6.0–12.2)	<0.001
	Glucose, mg/dL	262 (186–331)	249 (187–305)	274 (186–349)	0.310	254 (190–309)	274 (184–361)	0.418
	${\mathrm{PaO}}_{2}$, mmHg	143.0 (87.0–243.0)	125.5 (77.5–224.5)	163.5 (91.8–253.3)	0.076	138.0 (81.7–237.0)	162.0 (89.0–248.5)	0.452
	${\mathrm{PaCO}}_{2}$, mmHg	44.0 (34.0–63.0)	38.0 (32.8–44.0)	51.9 (35.8–70.0)	<0.001	38.9 (32.9–49.0)	53.5 (37.8–70.0)	<0.001
	SOFA score	11 (10–13)	10 (9–12)	12 (10–14)	<0.001	11 (9–12)	12 (10–14)	<0.001

CPR, cardiopulmonary resuscitation; ROSC, return of spontaneous circulation; ${\mathrm{PaO}}_{2}$, partial pressure of oxygen; ${\mathrm{PaCO}}_{2}$, partial pressure of carbon dioxide; SOFA, Sequential Organ Failure Assessment.

### 3.2 Baseline Characteristics According to Neurological Outcome and Mortality at 6 Months

Table 1 compares baseline characteristics between the neurological outcome and mortality groups. Compared to patients with a poor neurological outcome, patients with a good neurological outcome were younger and had lower incidence of comorbidities (hypertension and diabetes), a higher incidence of shockable monitored rhythm and cardiac etiology, shorter downtimes, lower lactate, and ${\mathrm{PaCO}}_{2}$ levels, and lower SOFA scores (Table 1). Compared with those who died, survivors were younger and had a higher incidence of bystander CPR and cardiac etiology, shorter downtimes, lower lactate, ${\mathrm{PaCO}}_{2}$ levels, and SOFA scores (Table 1).

### 3.3 Serum TnI Levels and TnI-C in OHCA Patients

Table 2 compares the TnI levels and TnI-C between neurological outcome and mortality groups. The TnI1st was higher in patients with a good neurological outcome than in patients with a poor neurological outcome (Table 2). There were no differences in the TnI levels between survivors and non-survivors. Survivors and patients with a good neurological outcome had a higher TnI1st compared with non-survivors and patients with a poor neurological outcome (Table 2). The proportion of patients with a TnI-C1st <50% was higher among non-survivors and those with a poor neurological outcome than in survivors and patients with a good neurological outcome (Table 2).

**Table 2.** **Comparisons of troponin-I levels according to neurological outcomes or mortality at 6 months**.

Variables	Total (n = 227)	Neurological outcomes at 6 months			Mortality at 6 months		
		Good (n = 77)	Poor (n = 150)	*p*	Survivors (n = 109)	Non-survivors (n = 118)	*p*
TnI1st, ng/mL	4.07 (0.88–19.53)	5.06 (1.84–29.96)	2.79 (0.77–13.94)	0.039	4.73 (0.98–25.73)	2.79 (0.84–11.95)	0.185
TnI2nd, ng/mL	1.72 (0.42–10.11)	2.84 (0.57–13.28)	1.45 (0.41–9.45)	0.215	2.47 (0.41–13.28)	1.46 (0.43–9.17)	0.475
TnI3rd, ng/mL	1.20 (0.24–7.80)	1.62 (0.28–9.98)	0.98 (0.21–7.40)	0.281	1.61 (0.24–9.98)	1.00 (0.24–7.54)	0.581
TnI-C1st, %	45.5 (23.1–62.6)	54.4 (33.6–71.0)	42.3 (17.3–57.7)	0.001	52.1 (30.1–65.9)	42.7 (17.3–55.9)	0.015
TnI-C1st <50% (%)	131 (57.7)	33 (42.9)	98 (65.3)	0.002	52 (47.7)	79 (66.9)	0.005
TnI-C2nd, %	37.0 (15.7–51.7)	43.0 (16.0–54.7)	35.9 (10.1–51.1)	0.306	40.5 (21.6–52.0)	32.4 (8.1–51.9)	0.170
TnI-C2nd <50% (%)	158 (69.6)	52 (67.5)	106 (70.7)	0.739	76 (69.7)	82 (69.5)	1.000

TnI, troponin-I; TnI-C, troponin-I clearance.

### 3.4 Association between TnI-C Clearance and Neurological Outcome or Mortality at 6 Months

Table 3 shows the association between TnI-C and poor neurological outcome or mortality at 6 months. Age, shockable monitored rhythm, time to ROSC, and ${\mathrm{PaCO}}_{2}$ were selected as the adjusted variables for poor neurological outcome at 6 months (**Supplementary Table 1**), and age, shockable monitored rhythm, and time to ROSC were selected as the adjusted variables for 6-month mortality (**Supplementary Table 2**). In a multivariable analysis, a TnI-C1st <50% was independently associated with both a poor neurological outcome (OR 0.371, 95% CI 0.169–0.819, *p* = 0.014) and mortality at 6 months (OR 0.469, 95% CI 0.245–0.898, *p* = 0.022). In contrast, neither TnI-C1st nor TnI-C2nd was related to either poor neurological outcome or mortality at 6 months.

**Table 3.** **Multivariable logistic regression analysis of troponin-I levels and poor neurological outcomes or mortality at 6 months**.

Variables	Neurological outcomes at 6 months		Mortality at 6 months	
	Adjusted OR ${\mathrm{(95\% CI)}}^{\text{a}}$	*p*	Adjusted OR ${\mathrm{(95\% CI)}}^{\text{b}}$	*p*
TnI1st, ng/mL	1.003 (0.994–1.012)	0.489	1.006 (0.998–1.015)	0.138
TnI2nd, ng/mL	1.008 (0.997–1.018)	0.158	1.009 (1.000–1.019)	0.060
TnI3rd, ng/mL	1.008 (0.997–1.020)	0.157	1.011 (1.000–1.022)	0.051
TnI-C1st, %	1.194 (0.602–2.368)	0.612	1.027 (0.614–1.718)	0.919
TnI-C1st <50% (%)	2.078 (1.080–3.995)	0.028	2.131 (1.114–4.078)	0.022
TnI-C2nd, %	1.479 (0.837–2.614)	0.178	1.098 (0.674–1.789)	0.707
TnI-C2nd <50% (%)	1.255 (0.623–2.528)	0.524	1.233 (0.615–2.469)	0.555

Each variable was individually entered into the final model and analyzed separately. ^a^Adjusted for age, shockable rhythm, time from collapse to return of spontaneous circulation, and ${\mathrm{PaCO}}_{2}$ level. ^b^Adjusted for age, shockable rhythm, and time from collapse to return of spontaneous circulation. OR, odds ratio; CI, confidence interval; TnI, troponin-I; TnI-C, troponin-I clearance; ${\mathrm{PaCO}}_{2}$, partial pressure of carbon dioxide.

### 3.5 Association between TnI-related Variables and Renal Impairment Due to Preexisting Illnesses

**Supplementary Table 3** shows the association between the TnI-related variables and the patients’ renal impairment due to a preexisting illness. The TnI1st, TnI2nd, TnI3rd, and TnI-C1st in patients without renal impairment were higher compared with patients with renal impairment. The proportion of patients with a TnI-C1st of <50% was lower in patients without renal impairment compared with patients with renal impairment.

## 4. Discussion

In this retrospective cohort study of OHCA patients treated with TTM, we found that the TnI-C1st after ROSC was lower in patients with a poor neurological outcome and in non-survivors at 6 months than in those with a good neurological outcome and survivors. After adjusting for potential confounders, TnI-C1st <50% was found to be a surrogate marker for a poor neurological outcome or mortality at 6 months.

In the present study, the TnI levels were higher than normal in most OHCA patients after ROSC. While previous studies have also reported elevated TnI levels after cardiac arrest [4, 6, 16], the peak TnI level exhibited at admission was lower than in the present study. The time to ROSC and the proportion of patients with a shockable rhythm during either arrest, witnessed arrest, or bystander CPR may help explain this difference, although this information was not available in every study. Various mechanisms can cause elevated TnI levels, such as secondary ischemic injury to the heart during cardiac arrest, the effects of defibrillation, acute myocardial infarction due to coronary artery occlusion, and cardiogenic shock after ROSC [17, 18, 19]. However, in OHCA patients, the relationship between elevated TnI levels and prognosis is controversial [6, 20]. In a single-center retrospective cohort study of OHCA patients, the peak TnI level was neither associated with in-hospital mortality nor poor neurological outcome at discharge [6]. Another study reported an association between high troponin levels and survival to discharge in OHCA patients with elevated ST [20]. A multivariable analysis in the present study showed that elevated TnI levels up to 3 days after ROSC were neither associated with poor neurological outcome nor mortality at 6 months in OHCA patients.

Although TnI-C1st did not have a linear relationship with the outcome of OHCA patients in the present study, a TnI-C1st <50% was associated with a poor neurological outcome and mortality at 6 months in OHCA patients. Originally, the TnI half-life is several hours [21]; however, in the case of an ongoing injury to the myocardium, such as a myocardial infarction, the TnI half-life is known to increase to approximately 24 hours [13]. Although TnI levels were elevated in OHCA patients because of the ischemic injury inflicted during cardiac arrest, an elevation in TnI levels was not associated with any outcomes. In other words, if the same cardiac arrest ischemic injury is applied to individual OHCA patients, the prognosis may vary depending on the patient’s body metabolism. Depending on the degree of metabolism during reperfusion injury, the TnI levels may decrease rapidly or slowly, or even increase. In the present study, this physiologic response may be indirectly expressed through TnI-C. The relationship between TnI-C and disease severity is shown in several severe conditions. Among patients with suspected acute coronary syndrome, the measurement of TnI 3 hours after admission may facilitate the early ruling-out of an acute myocardial infarction [22]. Consistently elevated TnI levels were associated with 90-day mortality and re-admission in patients with heart failure [23], and a consistently high TnI level after 24 hours was associated with mortality in patients who had coronary artery bypass surgery [24]. In patients with chronic renal failure, low troponin clearance was associated with one-month mortality [11]. In contrast, TnI-C2nd was not associated with any outcomes, probably because the reperfusion injury was most active up to 1 day after ROSC [25].

In the present study, TnI1st was higher in patients with a good neurological outcome compared with patients with a poor neurological outcome; a similar trend was observed in survivors and non-survivors. It may seem paradoxical that the TnI levels, which are related to myocardial ischemia, were higher in the good outcome group since TnI is specific for coronary artery disease [26]. Previous studies have shown that patients whose OHCA has a cardiac origin, including coronary artery disease, have a better prognosis than patients whose OHCA is non-cardiac in origin [27]. Simply, it is not possible to determine OHCA severity or prognosis using a TnI level taken at a single time point instead of examining changes in the TnI level. The TnI-C can help select patients who may need more intensive treatment by identifying their OHCA severity.

In this study, patients with renal impairment due to a preexisting illness had a lower TnI-C compared with patients without renal impairment. Previous studies have shown that the TnI values frequently rise and fall slowly in patients with renal disease, even those without coronary artery disease [28]. However, we do not believe that renal impairment due to a preexisting illness influenced this study’s findings because for renal impairment to affect the TnI-C, the peak TnI value must also be high. However, in this study, patients with renal impairment had lower peak TnI values than those without, making it difficult to conclude that renal impairment was affected. In addition, renal impairment due to a preexisting illness was not related to a poor neurological outcome or mortality of patients at 6 months post-OHCA.

## 5. Limitations

This study has several limitations. First, as this was a single-center retrospective observational study, multicenter studies with larger sample sizes are warranted to assess generalizability and causality. Second, our study did not analyze TnI levels immediately after ROSC since blood sampling was delayed because of patients being transferred after treatment at another hospital or initial intensive treatment. Third, although we tried, we could not measure TnI at the designated time in our study, making it impossible to confirm the relationship between changes in TnI level over time and patient prognosis. However, because TnI levels were collected within a maximum of 6 hours from the designated time and presented through the peak value, the associated error is not expected to be substantial. Fourth, there were three patients whose TnI could not be measured. Although this number was small, selection bias may have occurred because these patients were excluded. Fifth, the OHCA patients who did not undergo TTM were excluded from this study. Most of the OHCA patients who did not undergo TTM were transferred to other hospitals without being hospitalized at our hospital, and their long-term prognosis could not be accurately measured. Therefore, we decided to exclude the OHCA patients who did not undergo TTM from this study. Sixth, although we investigated renal impairment due to a preexisting illness and TnI-related variables, these relationships do not fully explain the effect of renal impairment on the TnI-related variables in OHCA patients. Furthermore, this study did not investigate whether an acute kidney injury occurred post-resuscitation or if continuous renal replacement therapy was administered. Thus, further research is needed to clarify this in the future. Finally, 11 patients were withdrawn from the study because of interrupted TTM after transfer or death, which may have led to selection bias.

## 6. Conclusions

In OHCA patients, a TnI-C1st <50% after ROSC was associated with poor neurological outcome and mortality at 6 months.

## Data Availability

All data generated or analyzed during this study are included in this article and its supplementary material files. Further inquiries can be directed to the corresponding author.

## Supplementary Material

Supplementary material associated with this article can be found, in the online version, at https://doi.org/10.31083/j.rcm2501024.
