# Supplementary material for: The Association between Troponin-I Clearance after the Return of Spontaneous Circulation and Outcomes in Out-of-Hospital Cardiac Arrest Patients
Source: Rev Cardiovasc Med. 2024 Jan 15;25(1):24. doi: 10.31083/j.rcm2501024 (PMC11262335; doi:10.31083/j.rcm2501024)
Supplement: Supplementary file 1 [file 2153-8174-25-1-024-s1.docx]

Supplementary Table 1. Multivariable logistic regression analysis of poor neurological outcome at 6 months.

|  | Adjusted OR (95% CI) | P |
| --- | --- | --- |
| Age, year | 1.057 (1.028–1.087) | < 0.001 |
| Hypertension | 1.366 (0.554–3.367) | 0.498 |
| Diabetes | 2.238 (0.901–5.560) | 0.083 |
| Renal impairment | 1.310 (0.232–7.405) | 0.760 |
| Witnessed collapse | 0.957 (0.416–2.202) | 0.919 |
| Shockable rhythm | 0.258 (0.095–0.696) | 0.007 |
| Cardiac etiology | 0.371 (0.122–1.129) | 0.081 |
| Time to ROSC, min | 1.055 (1.027–1.083) | < 0.001 |
| Lactate, mmol/L | 1.082 (0.985–1.188) | 0.099 |
| PaCO_2_, mmHg | 1.027 (1.002–1.052) | 0.035 |
| SOFA score | 1.008 (0.845–1.203) | 0.930 |

OR = odds ratio, CI = confidence interval, ROSC = return of spontaneous circulation, PaCO_2_ = partial pressure of carbon dioxide, SOFA = Sequential Organ Failure Assessment.

Supplementary Table 2. Multivariable logistic regression analysis for 6-month mortality.

|  | Adjusted OR (95% CI) | P |
| --- | --- | --- |
| Age, year | 1.027 (1.006–1.049) | 0.010 |
| Hypertension | 1.110 (0.541–2.279) | 0.776 |
| Diabetes | 1.012 (0.456–2.245) | 0.977 |
| Witnessed collapse | 0.842 (0.409–1.733) | 0.640 |
| Bystander CPR | 0.609 (0.311–1.194) | 0.149 |
| Shockable rhythm | 0.168 (0.085–0.331) | < 0.001 |
| Cardiac etiology | 0.722 (0.306–1.700) | 0.456 |
| Time to ROSC, min | 1.034 (1.015–1.053) | < 0.001 |
| Lactate, mmol/L | 1.050 (0.979–1.127) | 0.173 |
| Glucose, mg/dL | 0.999 (0.996–1.001) | 0.296 |
| PaCO_2_, mmHg | 1.016 (1.000–1.033) | 0.055 |
| SOFA score | 1.072 (0.929–1.236) | 0.342 |

OR = odds ratio, CI = confidence interval, CPR = cardiopulmonary resuscitation, ROSC = return of spontaneous circulation, PaCO_2_ = partial pressure of carbon dioxide, SOFA = Sequential Organ Failure Assessment.

Supplementary Table 3. Comparisons of troponin-I levels with renal impairment due to preexisting illness.

| Variables | Patients without renal impairment (n = 206) | Patients with renal impairment (n = 21) | P |
| --- | --- | --- | --- |
| TnI1st, ng/mL | 4.42 (1.00–21.24) | 0.78 (0.25–1.76) | 0.001 |
| TnI2nd, ng/mL | 2.14 (0.54–10.84) | 0.56 (0.20–1.59) | 0.006 |
| TnI3rd, ng/mL | 1.39 (0.28–8.52) | 0.32 (0.10–1.62) | 0.019 |
| TnI-C1st, % | 47.1 (24.5–63.2) | 38.3 (10.0–47.3) | 0.032 |
| TnI-C1st < 50% (%) | 113 (54.9) | 18 (85.7) | 0.013 |
| TnI-C2nd, % | 38.4 (16.0–51.9) | 21.6 (6.3–48.5) | 0.121 |
| TnI-C2nd < 50% (%) | 142 (68.9) | 16 (76.2) | 0.660 |

TnI = troponin-I, TnI-C = troponin-I clearance.
